# Interaction between Chinese medicine and digoxin: Clinical and research update

**DOI:** 10.3389/fphar.2023.1040778

**Published:** 2023-02-07

**Authors:** Wei Zhuang, Tao He, Bei-Bei Jia, Zhi-Zhou Wang, Lan Zhang, Xian-Zhe Dong, Sheng-Yan Xi

**Affiliations:** ^1^ Department of Pharmacy, Xuanwu Hospital of Capital Medical University, National Gerontic Disease Clinical Research Center, Beijing, China; ^2^ Department of Pharmacy, Eye Hospital China Academy of Chinese Medical Sciences, Beijing, China; ^3^ Department of Traditional Chinese Medicine, School of Medicine, Xiamen University, Xiamen, China

**Keywords:** traditional Chinese medicine, interaction, plasma concentration, area under the curve, digoxin

## Abstract

**Background:** Digoxin is one of the most widely and commonly used cardiac drug, which plays an irreplaceable role in treating heart failure and arrhythmia. *The 2010 Edition of Pharmacopoeia of the People’s Republic of China* stipulates that the effective range of digoxin plasma concentration is 0.5–2.0 ng/mL and it is toxic at plasma concentration >2 ng/mL. Its effective plasma drug concentration is close to the toxic concentration, and large individual differences in the effects of the drug have been observed. It is often used in combination with other drugs, but drug interactions have a great impact on the plasma concentration of digoxin and lead to adverse reactions (ADRs), such as poisoning. Most of the reported drug interactions are with Western drugs. However, there are many combinations of traditional Chinese medicine (TCM) and Western drugs, TCM interacting with digoxin comprises monomer components, single medicines, and Chinese patent medicines.

**Aim of the study:** We aimed i) to provide an overview of the TCM formulations affecting the pharmacology of digoxin and their mechanisms of action and ii) to provide a theoretical reference for the safe and rational use of digoxin in combination with TCM in clinical practice and to avoid ADRs.

**Methods:** A literature search of electronic databases, including PubMed, MEDLINE, Cochrane Library, Web of Science, China National Knowledge Infrastructure, and WANFANG Data, was performed to search for articles published between 1 January 1960, and 1 August 2022. Search terms used included “digoxin,” “traditional Chinese medicine,” “Chinese patent medicine,” and “adverse reactions” and their combinations.

**Results:** A total of 49 articles were obtained, including clinical reports, pharmacological experiments and *in vitro* experiments. The mechanisms of action affecting the pharmacology of digoxin are complex. TCM formulations may affect the pharmacology of digoxin *in vivo* by influencing gastrointestinal motility or gastric juice pH, regulating P-glycoprotein levels, exerting cumulative pharmacological effects, and enhancing the sensitivity of the heart to digoxin. Although studies have shown that some TCM formulations interact with digoxin, they may be influenced by the complexity of the composition and the pharmacological effects of the TCM, the sensitivity of digoxin concentration determination methods, etc. The results of existing studies are controversial and further in-depth studies are required.

**Conclusion:** Combinations of digoxin and TCM formulations are commonly used. This article serves as a reference to understand the interactions between TCM formulations and digoxin to avoid the occurrence of ADRs and improve the efficacy and safety of digoxin.

## Introduction

Digoxin is a pure preparation derived from the leaves of *Digitalis lanata* Ehrh (Maohuayangdihuang). It is a cardiac glycoside commonly used in clinical practice for the treatment of heart failure, atrial fibrillation, and other arrhythmias (Srinivasan et al., 2020). There is significant individual variation in patient response to digoxin therapy, and an important reason for this variation is that the disposition and sensitivity of patients to the drug vary greatly.

Digoxin shows large individual variation *in vivo*. It has a narrow therapeutic window, i.e., the therapeutic concentration is close to the toxic concentration; its effective plasma concentration is 0.5–1.5 ng/mL and it is toxic at plasma concentration >2 ng/mL. Moreover, its efficacy is influenced by several factors (Liu et al., 2016), including drug interactions. Digoxin is one of the 10 drugs with the most drug interactions, and the incidence of adverse reactions (ADRs) due to interactions is as high as 27% (Sun, 1982). Most of these reports and studies are on combinations with Western drugs. In this paper, we summarize the interactions of digoxin with other drugs and their mechanisms of action from a pharmacological point of view by summarizing basic research, clinical studies, treatment guidelines and monographs, to provide a reference for its safe clinical use.

## Methods

A literature search of electronic databases, including PubMed, MEDLINE, Cochrane Library, Web of Science, China National Knowledge Infrastructure, and WANFANG Data, was performed to search for articles published between 1 January 1960 and 1 August 2022. Search terms used included “digoxin”, “Chinese medicine”, “traditional Chinese medicine”, “Chinese patent medicine”, “adverse reactions” and their combinations. The inclusion criteria included the following: a. Clear literatures on the experimental and clinical research on the interactions between TCM formulations and digoxin. b. Literatures written in English and Chinese. The exclusion criteria were as follows: a. Literatures without mechanisms of interactions between TCM formulations and digoxin; b. Literature review, expert experience introduction and other types of literature; c. For the content of the repeatedly published literature. A total of 49 articles were obtained, including clinical reports, pharmacological experiments and *in vitro* experiments which were reviewed to determine the mechanism of interactions between TCM and digoxin.

## Results

### The pharmacology of digoxin

#### The pharmacokinetic process of digoxin

Oral and injectable digoxin formulations exist. Because the injection is prone to ADRs and is only suitable for patients with severe heart failure requiring immediate treatment, oral formulations are more frequently used. Thus, this article focuses on the pharmacokinetic processes of oral formulations. Digoxin is absorbed orally through the small intestine, with incomplete and irregular absorption. The absorption rate is about 75%, the onset of action is at 0.5–2 h, the peak plasma concentration is observed at 2–3 h, and the maximum effect is observed at 4–8 h. After absorption, it is widely distributed to various tissues, such as the heart, bone, muscle, liver, and kidney (Bai et al., 2014). It is partly absorbed into the blood *via* the biliary tract, forming hepatic-intestinal circulation, with a low plasma protein binding rate of 20%–25% and an apparent volume of distribution (V_d_) of 6–10 L/kg. The elimination half-life period (t_1/2_) of digoxin with normal renal function is 36 h on average, and the steady state can be reached after 5 ×*t*_1/2_ (about 7 days). The bioavailability of oral digoxin is 60%–80% (Yang et al., 2019).

### Influencing the pharmacokinetics of digoxin

#### Gastrointestinal factors affect the absorption process

When digoxin is used in combination with gastrointestinal stimulants, gastrointestinal stimulants may increase the clearance of digoxin because they promote gastric emptying and intestinal peristalsis; changes in the pH value of the gastrointestinal tract also affect the absorption of digoxin (Li and Pan, 2011)*.* In addition, some bacteria present in the gastrointestinal tract may affect the pharmacokinetic parameters of digoxin. For example, digoxin can be partially metabolized by intestinal anaerobic bacteria, including Bifidobacterium after oral administration of non-cardiogenic dihydrodigoxin and dihydrodigoxin sapogenins, which decreases the plasma concentration of digoxin (Bai et al., 2014).

#### Drug transporters

Drug transporters are among the main factors affecting the *in vivo* disposal of drugs. P-glycoprotein (P-gP), which belongs to the superfamily of ATP-binding cassette (ABC) transporters, is the main efflux protein in humans. P-gP is mainly expressed on capillary endothelial cells in the small intestine, liver, kidney, and blood-brain barrier (Thiebaut et al., 1987). Its main function is to excrete intracellular toxic substances and exogenous substances such as drugs through the gastrointestinal tract, bile, or urine. Since the activity of P-gP can affect the plasma concentration, bioavailability, tissue distribution, and liver and kidney clearance rate (CL) of the drug (Herfindal et al., 2011), during co-administration, certain drugs can act as substrates, P-gP inhibitors, or P-gP inducers, causing changes in the plasma concentration of the co-administered drugs leading to interactions. Digoxin is a substrate of P-gP. P-gP mediates the renal excretion of digoxin while promoting the absorption process in the small intestine (Mamidi, et al., 2017). When digoxin is combined with drugs such as P-gP inhibitors or P-gP inducers, changes in the pharmacokinetic parameters of digoxin may be caused by altered P-gP expression or activity in the intestinal wall, thus altering the bioavailability of digoxin (Li et al., 2011).

### The pharmacodynamics of digoxin

Digoxin belongs to cardiac glycosides and has a positive inotropic effect. It acts directly on myocardial cells, inhibits Na^+^/K^+^-ATPase activity by selectively binding with Na^+^/K^+^-ATPase on the myocardial cell membrane, impairs Na^+^-K^+^ active coupling transport inside and outside the myocardial cell membrane, increases Na^+^ concentration in myocardial cells, makes Na^+^ and Ca^2+^ exchange on myocardial membrane tend to be active, increases Ca^2+^ in myocardial cells, activates the myocardial contractile protein, increases myocardial contractility, and produces a cardiotonic effect (Jiri et al., 2020).

### Influencing the pharmacodynamics of digoxin

#### Hypokalemia

Low blood potassium level is the most important factor affecting digoxin toxicity (Du et al., 2016). Blood potassium levels are positively correlated with cardiomyocyte potassium channel permeability, and low potassium levels inhibit cardiomyocyte potassium outflow, leading to increased cardiac autoregulation and inducing cardiac arrhythmias caused by cardiac glycosides.

### Special populations

Endogenous digoxin substances are present in neonates and patients with renal dysfunction, and these substances may lead to elevated or false-positive digoxin plasma concentration monitoring results. Patients with higher body weight have a large V_d_ of digoxin and relatively low digoxin plasma concentration (Du et al., 2016).

### Genes and ethnicity

Pharmacogenomic studies of digoxin revealed that polymorphisms at certain loci can affect the metabolism and action of digoxin. For example, digoxin plasma concentration in patients with 3435 TT (3.74 ± 1.06 ng/mL) and 2677 GG (3.40 ± 1.35 ng/mL) phenotypes is significantly higher than in patients with 3435CC/CT (0.79 ± 1.06 ng/mL/1.24 ± 0.66 ng/mL) and 2677TT/TG (1.31 ± 1.20 ng/mL/1.47 ± 1.16 ng/mL) genotypes, further suggesting that ABCB1 gene polymorphisms can affect digoxin plasma concentration (Jiang et al., 2019), thus explaining the uncommon effective digoxin dose in some patients. Yan et al. (2015) studied the clinical effects and differences in plasma concentration of digoxin in the treatment of Uyghur and Han Chinese heart failure patients. Their results showed that the efficacy of digoxin in the treatment of heart failure in Uyghur and Han Chinese was comparable, but digoxin plasma concentration was significantly higher in the Han Chinese group (1.41 ± 0.43 ng/mL) than in the Uyghur group (0.96 ± 0.39 ng/mL).

### Analysis of the interactions between TCM formulations and digoxin and the underlying mechanisms

**FIGURE 1:**
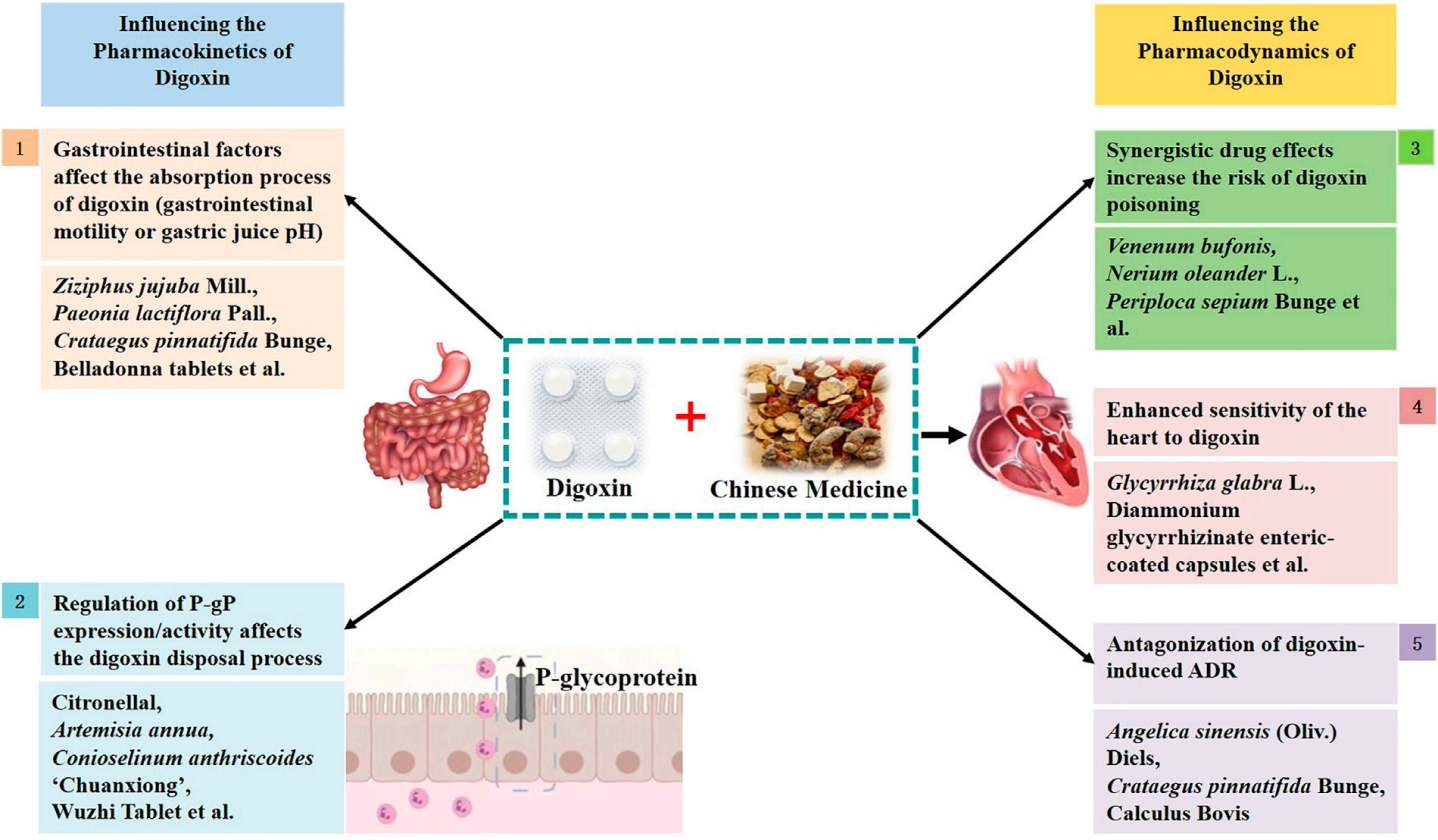
Schematic diagram of the mechanism of action between digoxin and traditional Chinese medicine.

Based on our analysis of the literature, the mechanism of interaction between TCM formulations and digoxin is as follows: A. Effect of TCM on pharmacokinetic mechanism of digoxin i) they can influence the state of the gastrointestinal tract and affect digoxin absorption; ii) they can modulate P-gP expression/activity and thereby influence digoxin disposal; B. Effect of TCM on Pharmacodynamics mechanism of digoxin iii) synergistic drug effects may occur, increasing the risk of digoxin poisoning; iv) they may enhance the sensitivity of the heart to digoxin and increase the risk of poisoning; and v) they may antagonize the ADRs caused by digoxin (Figure 1).

## Effect of TCM on pharmacokinetic mechanism of digoxin

### The status of the gastrointestinal tract and digoxin absorption

#### Reduced gastrointestinal motility

Anticholinergics can decrease intestinal peristalsis, prolong the passage time of digoxin in the upper part of the small intestine, increase digoxin absorption, and increase bioavailability (Qiu., 2016)*.* TCM formulations containing tropane alkaloids, such as belladonna tablets, increase the absorption and accumulation of cardiac glycosides in muscle tissue because of the relaxing effect of tropane alkaloids on smooth muscle tissue and reduced gastrointestinal motility, especially in patients with heart failure who are sensitive to the effects of cardiac glycosides and prone to toxic reactions.

#### Influence on gastric juice pH

A study on the effect of gastric juice pH on the biotransformation of digoxin was carried out. Two groups were given gastrin and normal saline respectively in addition to ^3^H-labeled digoxin. After 90 min, the content of digoxin in gastric juice was determined. The results showed that the content of digoxin in the normal saline group (52.5%) was significantly higher than that in the gastrin group (12.5%) which indicated that inappropriate pH of gastric juice can destroy digoxin. The transformation of digoxin *in vivo* requires a suitable pH environment, and peracid or peralkaline may affect the absorption of digoxin (Gault et al., 1980). Increased gastric acid secretion and lower gastric juice pH can increase the hydrolysis of digoxin-to-digoxin sapogenins and increase the metabolite levels in urine (Gault et al., 1981)*.* Thus, gastric acid has a destructive effect on digoxin and can reduce digoxin bioavailability. In addition, ADRs of the digestive system are the most common adverse effects of cardiac glycosides, often involving nausea and vomiting (Mladěnka et al., 2018). Studies have shown that digoxin can cause a significant decrease in gastric juice pH in mice and gastric and duodenal congestion, and the effects of oral administration are more severe than those of intravenous administration, further suggesting that digoxin can induce peptic ulcers (Fan et al., 2018), so attention should be paid to digestive system reactions caused by combinations of TCM formulations with digoxin that can cause a decrease in gastric juice pH. Common TCM formulations that can cause a decrease in pH include those containing large amounts of tannins, such as *Ziziphus jujuba* Mill. (Suanzaoren), *Paeonia lactiflora* Pall. (Baishao), *Crataegus pinnatifida* Bunge (Shanzha), *Prunus mume* (Siebold) Siebold and Zucc. (Meihua), *Senegalia catechu* (L.f.) P.J.H. Hurter and Mabb. (Ercha), *Rosa laevigata* Michx. (Jinyingzi), *Schisandra chinensis* (Turcz.) Baill. (Wuweizi), *Taraxacum mongolicum* Hand.-Mazz (Pugongying), *Cornus officinalis* Siebold and Zucc. (Shanzhuyu), *Ligustrum lucidum* W.T. Aiton (Nüzhenzi), *Rheum officinale* Baill. (Dahuang), *Agrimonia pilosa* Ledeb (Xianhecao), *Citrus aurantium* L. (Zhishi), *Lonicera japonica* Thunb. (Jinyinhua), and *Chaenomeles lagenaria* (Loisel.) Koidz (Mugua), whose aqueous solutions are acidic due to the phenolic hydroxyl group in their structures (Qi et al., 2014), and Chinese patent medicines such as Shanzha Pill, Baohe Pill, Wumei Pill, and Wuweizi Pill. *In vitro* experiments have confirmed that antacids such as magnesium trisilicate, which elevate the pH of gastric juice, affect the absorption of digoxin, thus, decreasing the bioavailability of digoxin (Khalil, 1974).

#### Others

Wheat bran is the seed coat of the grass plant wheat, which harmonizes the middle and benefits the spleen (Wu et al., 2014). Wheat bran itself has little effect on digoxin, but if ingested at the same time as digoxin, it may form a bond with digoxin, which affects the absorption of digoxin and reduces digoxin plasma concentration (31.2% less than digoxin alone), thus, reducing the bioavailability of digoxin (Reissell and Manninen, 1982).

### Regulation of P-gP affects the digoxin disposal process

#### Downregulation or inhibition of P-gP

*TCM extracts*: A study on the effect of citronellal on intestinal absorption of digoxin *in vivo* and *in vitro* was carried out. *In vivo*, digoxin was administered intravenously to control rats and citronellal pretreated experimental rats, and *in vitro*, the transcellular transport of ^3^H-digoxin across Caco-2 cell monomolecular membrane was measured in the presence or absence of citronella. The results have demonstrated that monoterpenoids such as citronellal, derived from *Zanthoxyli Fructus*, can increase the bioavailability (from 75.8% of digoxin alone to 99.9% of digoxin combined) by inhibiting P-gP, but have no effect on its CL and V_d_ (Yoshida et al., 2006) of digoxin. Flavonoids such as nobiletin, quercetin, lanosterin and cathepsin can inhibit P-gP activity or suppress P-gP expression, thus inhibiting digoxin excretion, increasing plasma concentration of digoxin and increasing bioavailability. Studies in MDR1-MDCK II cells confirmed that nobiletin can inhibit P-gP activity and thus inhibit digoxin. *In vivo* pharmacokinetic experiments in rats showed that nobiletin could increase the area under the curve (AUC) and the maximum plasma concentration (C_max_) of digoxin 2.02-fold and 3.29-fold, respectively, and molecular docking simulations between nobiletin and P-gP suggested that nobiletin forms a strong π-π bond, which may be an important factor in the inhibitory effect (Bai et al., 2018). A study using pigs as experimental subjects showed a very strong pharmacokinetic interaction between quercetin and digoxin. If 0.02 mg/kg digoxin was combined with 40 mg/kg quercetin, this increased the C_max_ of digoxin by 413% and the AUC by 170%; if 0.02 mg/kg digoxin was combined with 50 mg/kg quercetin after 30 min, this could cause death (Wang et al., 2004). Jin et al. (2015) found that lanosterin was able to inhibit P-gP expression in the brain, and if combined with digoxin, it may elevate the plasma concentration of digoxin. In a study to evaluate the inhibitory effect of cathepsin on P-gP transport activity in the presence or absence of artemisinin, digoxin, a specific substrate of P-gP in rat plasma, was quantitatively analyzed by UHPLC-MS/MS method. The results have shown that cathepsin, one of the polymethoxylated flavonoids in *Artemisia annua* (Qinghao), is able to inhibit P-gP activity and non-competitively inhibit CYP3A, which can effectively inhibit CYP3A-induced metabolism and P-gP-mediated transport and has a significant effect on the P-gP substrate digoxin, increasing AUC (2.34 times) and C_max_ (1.81 times) and decreasing CL (3.50 times); however, catnip flavonols significantly inhibited P-gP-mediated digoxin activity when combined with *A. annua* (Qinghao) in a 1:2 ratio, while *A. annua* (Qinghao) alone had no significant effect (Yang et al., 2016). Ginsenosides can competitively bind P-gP sites, and if combined with digoxin, they can i) inhibit the transport of digoxin by P-gP and additionally ii) compete with digoxin for P-gP binding sites, leading to an increase in plasma concentration of free digoxin (Li et al., 2014; Wang, 2019).

*Single TCM:* Single TCM formulations that are capable of inhibiting or downregulating P-gP include *Conioselinum anthriscoides* “Chuanxiong” (Chuanxiong), *Vernonia amygdalina* (Biantaobanjiuju), *Tapinanthus sessilifolius*, *Carica papaya* (Mugua) and *Ginkgo biloba* L. (Yinxing). In addition to inhibiting P-gP expression by affecting intracellular Ca^2+^ concentrations, *C. anthriscoides* “Chuanxiong” (Chuanxiong) can also increase the plasma concentration of digoxin by affecting the transport of digoxin in a monolayer model of Caco-2 cells (Feng et al., 2018) and by acting as a P-gP substrate (Yang et al., 2013), binding to P-gP, thereby decreasing the digoxin binding efficiency and thus increasing the plasma concentration of digoxin, and the main component of the interaction is chuanxiongzine (Yang et al., 2013). Japanese studies *in vivo* and *in vitro* using rats and cells as research subjects (Oga et al., 2012; Oga et al., 2013) have shown that the effects of *V. amygdalina*, *T. sessilifolius*, and *C. papaya* extracts had a significant inhibitory effect on P-gP expression, increasing digoxin absorption in the intestine, and in combination with digoxin they altered the pharmacokinetic distribution of digoxin, resulting in a significant increase or even a 2-fold increase in its AUC. A study by the University of Mississippi in the United States showed that *G. biloba* L. (Mauro et al., 2003) has no significant effect on pharmacokinetic parameters such as AUC, C_max_, t_1/2_, and CL of digoxin in humans. However, with the technological advancement, a new liquid chromatography tandem mass spectrometry (LC-MS/MS) method with higher sensitivity and reproducibility than the conventional assay was developed for the determination of *G. biloba* L. (Yinxing) extract in rat plasma by applying the dynamic multireaction monitoring system. When *G. biloba* L. (Yinxing) extract and digoxin are combined, *G. biloba* L. (Yinxing) extract had a significant effect on the pharmacokinetic parameters of digoxin, significantly increasing plasma concentration (1.25 times) and AUC (1.28 times), probably due to the inhibition of intestinal P-gP activity by flavonoids in *G. biloba* L. (Yinxing) extract (Rao et al., 2014).

*Chinese patent medicine and decoction:* Wuzhi Tablet is an ethanolic extract preparation of *S. chinensis* (Turcz.) Baill. (Wuweizi), which is a commonly used hepatoprotective drug (Xin et al., 2011). In combination with digoxin, *S. chinensis* (Turcz.) Baill. (Wuweizi) methyl, ethyl, alcohol B, and ester A in Wuzhi Tablet can increase the plasma concentration of digoxin, respectively from 22.3 ± 13.3 ng h/mL to 34.4 ± 23.4, 37.0 ± 14.2, 41.2 ± 15.1, and 39.0 ± 19.1 ng h/mL, among which the effect of *S. chinensis* (Turcz.) Baill. (Wuweizi) alcohol B is the strongest. Experiments in mice have shown that the AUC of digoxin is increased by gavage and intravenous injection. The AUC values of digoxin by gavage and intravenous injection were similar, suggesting that the plasma concentration of digoxin was increased mainly by inhibiting the clearance of digoxin in the liver and kidney. Cellular experiments showed that pentosidine, ethosidine, alcohol B, and ester A could inhibit the transport of digoxin in Caco-2 cells, suggesting that the above components could inhibit the activity of P-gP *in vitro*. In conclusion, Wuzhi Tablets elevate the plasma concentration of digoxin by inhibiting P-gP activity in the liver and kidney and reducing its elimination (Tan et al., 2018). Shaoyaogancao decoction is described in *Zhang Zhongjing’s Treatise on Febrile Diseases*. It consists of two herbs, *Paeonia lactiflora* Pall (Baishao) and *Glycyrrhiza glabra* L. (Gancao). When combined with digoxin, it has the potential to promote digoxin absorption and increase plasma concentration and thus bioavailability by downregulating P-gP expression and inhibiting P-gP function (Wang et al., 2012).

#### Upregulation of P-gP

Some studies suggest that TCM formulations with induction effects on P-gP include silymarin, auraptene, *Bupleurum chinense* DC. (Chaihu), *Cyperus rotundus* L. (Fuzi) and *St. John’s* wort (Guanyelianqiao). Silymarin significantly induces mRNA expression of P-gP and may decrease the bioavailability of digoxin and reduce its efficacy (Wang, 2019), but a study from the University of Mississippi showed no significant effect of silymarin on pharmacokinetic parameters such as AUC, C_max_, t_1/2_, and CL of digoxin in humans (Gurley et al., 2006). The micromolecular hydrophilic extract from vinegar-baked Radix *Bupleurum chinense* DC. (MHE), a water-soluble part of vinegar-baked Radix Bupleuri aqueous extract with polysaccharides removed by 80% *n*-butanol extraction, a study on the effect of MHE on the behaviour of digoxin, a P-gP substrate, in rats and its material basis has demonstrated that MHE significantly affected the pharmacokinetic behavior of digoxin (a decrease in AUC, an increase in CL/F), AUC of low dose MHE combined with digoxin decreased by 31.54% and CL/F increased by 29.6%. AUC of high dose MHE combined with digoxin decreased by 42.98%, CL/F increased by 59.02% as well as *in vivo* distribution (increased distribution in the liver, decreased distribution in the small intestine and blood, without affecting cardiac and renal drug distribution), and these changes were correlated with the amount of tissue P-gP expression (Xiao, 2016). The *Cyperus rotundus* L. (Fuzi) aqueous extract can upregulate the expression of P-gP in intestinal tissues at the mRNA and protein levels and thus may interact with digoxin, and the dose of the drug may be considered to be adjusted accordingly in clinical co-administration (Ji et al., 2019). *St. John’s* wort is a popular antidepressant herb; it could increase the clearance of certain drugs by modulating the P-gP efflux pump (Dasgupta, 2008), induce the production of intestinal P-gP, and stimulate the efflux of digoxin to the intestine, resulting in a decrease in plasma concentration (36%) of digoxin, a decrease in AUC (25%), and reduced efficacy (Cheng, 2000; Gurley et al., 2008). A study of possible pharmacokinetic interactions of *St. John’s* wort preparations and doses with digoxin showed that *St. John’s* wort preparations given as traditional gibberellin-containing products at daily doses of 0.5 or 1 g had no significant interaction with digoxin; however, in combination with high-dose gibberellin extract LI 160 or a control containing 4 g hypericin, the AUC of digoxin was reduced and the C_max_ was decreased, demonstrating the interaction between *St. John’s* wort and digoxin was closely related to the dose of hypericin (Mueller et al., 2004). Auraptene is a natural compound widely found in citrus fruits. Auraptene was shown to induce drug efflux by the P-gP transporter in human intestinal cells (Nabekura et al., 2020).

#### Others

Homology of medicine and food means that food and medicine have the same efficacy and no clear boundary in regulating human physiological function, and have the homology of source, composition and theory (Jia et al., 2021). Medicinal and edible homologous varieties interacting with digoxin include Citrus juices, Purple grape, Green tea and *Areca catechu* L. (Dasuan). Citrus juices such as orange juice, lemon juice, grape juice, and grapefruit juice contain a variety of flavonoid components with similar modulatory activity on digoxin transport as P-gP inhibitors, but the strength and characteristics of the effects depend on the distribution and classification of the flavonoid components (Xu et al., 2003). Purple grape is widely used for its health effects such as antioxidant activity, free radical scavenging, and anti-aging effects, and a study on the effect of purple grape on the pharmacokinetic parameters of digoxin in rats have confirmed that purple grape juice can inhibit P-gP in the gastrointestinal tract wall, and when used in combination with digoxin, it can increase pharmacokinetic parameters such as AUC (92.27%, 18.37 ± 1.67 ng h/mL to 35.32 ± 5.58 ng h/mL) and C_max_ (45%, 1.43 ± 0.15 ng/mL to 2.07 ± 0.36 ng/mL), thus increasing bioavailability, especially when multiple doses of purple grape juice are used, further suggesting that dose adjustment may be required when digoxin and purple grape juice are used together (Song et al., 2019)*.* Green tea polyphenols and catechins (Jodoin et al., 2002) inhibit P-gP but reduce human pharmacokinetic parameters such as AUC (8.20–5.44 ng h·mL^−1^) and C_max_ (2.47–1.76 ng/mL) of digoxin (Kim et al., 2018). *A. catechu* L. (Dasuan) induced human intestinal P-gP expression, which may cause digoxin efflux and decrease its bioavailability (Shi and klotz, 2012).

## Effect of TCM on pharmacodynamics mechanism of digoxin

### Synergistic drug effects increase the risk of digoxin poisoning

#### *Venenum bufonis* and preparations containing *venenum bufonis*

*Venenum bufonis* (Chansu) is the dried white pulp secreted from the skin glands and post-auricular glands of the Chinese toad or the black orbital toad, family Toadidae. The composition is complex, and the main components are toad toxin and toad venom ligands, all of which have the structure of cardiac glycosides of the toad steroidal dienolactone type, and animal experiments and clinical practice have proved that toad crisp, toad toxin, and toad venom ligands have cardiotonic effects, and their properties of action are similar to those of digitalis, but the cardiotonic effect is weaker (Dong, 2009; Chen, 2014). The crude venom of toad and one of its main components, toad venom ling, can enhance electrically driven contraction of isolated rat atria, constrict rat aortic rings, inhibit Na^+^/K^+^-ATPase in the rat aorta, and cross-react with anti-digoxin antibodies (Bagrov et al., 1993; Bressman et al., 2017). One of the components of toad parotid secretion, RI23, is a steroidal compound, mainly composed of toadoxigenin, and its digoxin-like effect on cardiotonic activity is mediated by blockage of voltage-dependent L-type calcium channels (Oliveira et al., 2020).

Commonly used Chinese patent medicines containing *Venenum bufonis* (Chansu) include Shexiangbaoxin Pill, Houtongxiaoyan Pill, Liushen Pill, Lushen Pill, and Jiuxin Pill. These compounds are classified as “toad venom dienolide,” which is similar to digoxin in chemical structure and therefore elicits a digoxin-like immune response and inhibits Na^+^/K^+^-ATPase (Dasgupta et al., 2002). Radioimmunoassay (RIA) was applied to detect Venenum bufonis (Zeng et al., 1992), and frequent premature ventricular contractions occurred after the combination of digoxin and Liushen Pill (Dong, 2009). Chinese patent medicines containing Venenum bufonis may interfere with the monitoring of digoxin plasma concentration. For example, when Jiuxin pills (KYUSHIA, Japan) were mixed into the serum, an abnormally high concentration of “digoxin” was detected in the supernatant (Yang and Xu, 2002).

#### *N. oleander* L.

*Nerium oleander* L. (Jiazhutao) is a common cause of poisoning in Southeast Asia. All parts of the *N. oleander* L. (Jiazhutao) shrub contain cardiac glycoside components that inhibit Na^+^/K^+^-ATPase, which may lead to oleander poisoning, causing symptoms similar to digoxin poisoning, including severe arrhythmia and even death. Current studies have shown that most of the cardiac glycosides of *N. oleander* L. (Jiazhutao) are much more toxic than digoxin (Amend et al., 2021).

#### *P. sepium* Bunge

*Periploca sepium* Bunge (Xiangjiapi) is widely used in Chinese medicine for the treatment of cardiac dysfunction, and its component periplocymarin has a strong cardiac glycoside structure with a strong affinity for Na^+^/K^+^-ATPase, which inhibits Na^+^/K^+^-ATPase activity by binding to Na^+^/K^+^-ATPase in the myocardial cell membrane, leading to increased Ca^2+^ inward flow, increased intracellular Ca^2+^ concentrations in the myocardium, and enhanced myocardial contractility, similar to the effects of digoxin (Yun et al., 2020).

#### *P. ginseng* C.A.Mey. and preparations containing *P. ginseng* C.A.Mey.

*Panax ginseng* C.A.Mey. (Renshen) can strengthen the heart, raise the blood pressure, improve coronary flow, increase the body’s ability to resist hypoxia, reduce myocardial oxygen consumption, protect and repair cardiomyocytes, and exert certain anti-arrhythmic effects. Some of its molecular structures are similar to those of digitalis glycosides, which are commonly used clinically in combination with digoxin for the treatment of coronary heart disease, heart failure, etc. The combined therapeutic effects can enhance each other. However, some studies have shown that *P. ginseng* (Renshen) does not affect the determination of serum digoxin (Chow et al., 2003). Shenmai injection promotes the renal excretion of digoxin and tends to decrease digoxin plasma concentration (Li et al., 2011). A study on the effect of Shenmai Injection on digoxin blood concentration and pharmacokinetic parameters in dogs with heart failure showed that there are differences in the pharmacokinetic effects of different doses on digoxin, all of which tend to shorten digoxin t_1/2_, but the low dose accelerates digoxin elimination in the blood with a lower AUC than the medium and high dose group and the digoxin control group, AUC of the low dose group, medium-dose group, high dose group and the digoxin control group respectively are 19.45 ± 4.27, 37.24 ± 10.72, 35.76 ± 7.39, and 35.64 ± 15.19 μg h/mL (Mao et al., 2010). The combination of Shenmai injection with digoxin for heart failure treatment does not lead to digoxin accumulation and does not increase the risk of digoxin toxicity (Mao et al., 2007).

#### *E. sinica* Stapf and preparations containing *E. sinica* Stapf

Ephedrine, the main component in *Ephedra sinica* Stapf (Caomahuang), has excitatory effects on cardiac α and β receptors, which excite the myocardium and enhance myocardial contractility. When combined with digoxin, *E. sinica* Stapf (Caomahuang) can enhance both the cardiotonic effects of digoxin and the toxic effects of digoxin on the heart, which can lead to toxic reactions including premature ventricular contractions, arrhythmia, and heart failure (Dong, 2009; Chen, 2014). Common Chinese patent medicines containing *E. sinica* Stapf include Jizhi syrup, Maxinzhike tablets, Tongxuanlifei pills, and Xiaokening tablets.

#### TCM formulations containing calcium

High serum Ca^2+^ concentrations can synergize with digoxin to enhance its effects, accelerate myocardial contractility, inhibit Na^+^/K^+^-ATPase, and increase digitalis toxicity, which may cause severe arrhythmia or even death, so combined use should be avoided (Dong, 2009). Experimental animal studies suggested that digoxin toxicity may be related to nuclear factor kappa-B (NF-κB), at least in part through regulation of the voltage-gated L-type calcium channel CaV1.2 and intracellular calcium homeostasis in cardiac myocytes, as NF-κB is an important transcription factor in most organ systems, often associated with cardiac injury, that can promote inflammatory responses and modulate cardiac function by regulating Ca^2+^ (Farghaly et al., 2018). In one case report, a patient who was given Qingkailing injection in combination with digoxin had nausea, precordial discomfort, a heart rate of 59 beats/min, and an electrocardiogram showing atrial fibrillation and second-degree atrioventricular block. After stopping digoxin and Qingkailing injection the symptoms disappeared with a heart rate of 88 beats/min and an electrocardiogram showing only atrial fibrillation. No similar symptoms occurred with the same dose of digoxin given alone later. It is believed that the toxic reaction of digitalis is directly related to the use of Qingkailing injection (Dong and Wang, 1993). The mechanism of digitalis toxicity induced by Qingkailing injection is not well understood; it may be related to the fact that mother-of-pearl in Qingkailing injection contains Ca^2+^, and the effect of Ca^2+^ on the heart is similar to that of digitalis, which can strengthen myocardial contraction, inhibit Na^+^/K^+^-ATPase, enhance the effects of cardiac glycosides and make them more toxic, and cause arrhythmia and conduction block, but the specific mechanism needs to be studied further (Dong, 2009).

Commonly used clinical single Chinese medicines containing calcium include Os Draconis (Longgu), Os Tigris (Hugu), Os Pardi (Baogu), antler (Lujiao), rhinoceros horn (Xijiao), Saigae Tataricae Cornu (Lingyangjiao), Bubali Cornu (Shuiniaojiao), magnetitum (Cishi), alumen (Baifan), Gypsum Fibrosum (Shigao), stalactitum (Zhongrushi), ophicalcitum (Huaruishi), fluoritum (Zishiying), hematite (Daizheshi), Natrii Sulfas (Mangxiao), calamina (Luganshi), talcum (Huashi), chalcanthitum (Danfan), Haliotidis Concha (Shijueming), Ostreae Concha (Muli), Margaritifera Concha (Zhenzhumu), ark shell (Walengzi), Concha Meretricis Seu Cyclinae (Haigeqiao), cuttlebone (Haipiaoxiao), pangolin scales (Chuanshanjia), Testudinis Carapacis Et Plastri (Guijia), Turtle Carapace (Biejia), *Lycium barbarum* L. (Gouqizi), lithargyrum (Mituoseng), and Crinis Carbonisatus (Xueyutan). Common Chinese patent medicines containing calcium include Qingkailing injection, Zhachongshisanwei Pills, Niuhuangjiedu Pill, Juhong Pills, Weitongning Pills, and Niuhuangshangqing Pills.

#### Qiliqiangxin capsules

The Qiliqiangxin capsule is a Chinese patent medicine for the treatment of chronic congestive heart failure (Chen and Pang, 2014). Its ingredients, *C. rotundus* L. (Fuzi) and *Descurainia sophia* (L.) Webb ex Prantl (Tinglizi), contain cardiac glycoside components, such as strophanthigenin, evomonoside, and helveticoside, which have different degrees of cardiac strengthening effects (Ma and Gao, 2004; Wang et al., 2013). When combined with digoxin it leads to increased digoxin plasma concentration, predisposes to digitalis toxicity, and is associated with patient age. The patients over 80 years old (27.5%) were more likely to be poisoned by the increase of digoxin concentration than those under 60 years old (15.30%), the proportion of digoxin poisoning in 60–80 years old patients (17.57%) was also higher than that under 60 years old (15.30%), but there was no statistical significance (Ye et al., 2019).

#### Others

TCM formulations containing cardiac glycosides, such as *Apocynum venetum* L. (Luobuma), *Tradescantia spathacea* Sw (Wannianqing), and *Salvia miltiorrhiza* Bunge (Danshen), when combined with cardiac glycosides such as digoxin, are prone to toxic reactions of digitalis-like cardiac glycosides due to the cumulative effect of the drug (Cheng, 2002; Dasgupta and Szelei-Stevens, 2004). If *A. venetum* L. (Luobuma) infusion or decoction contains cardiac components, both have obvious cardiac effects and are fast-acting cardiac glycosides, with pharmacological and toxic effects similar to digitalis (Zhai and Lv, 2009), and thus, it should not be used in combination. The cardiac glycosides isolated from *T. spathacea* Sw. (Wannianqing) have similar pharmacological effects as digitalis toxins, and the excitatory effect of the vagus nerve is 1.6 times stronger than that of digitalis toxins (Yu et al., 2012), which has direct inhibition and accumulation effects on the myocardium. A clinical case study reported the cardiotoxic reaction of a 16-year-old girl caused by an overdose (eight tablets) of Mexican Tejocote, which is marketed as a weight loss product with positive inotropic effects, the toxic reaction was similar to the cardiotoxic reaction of digoxin, and the heart rate returned to normal and the toxic reaction disappeared after 29 h of subsequent administration of digoxin immunization, so this weight loss product and digoxin in combination may elevate digoxin plasma concentration and lead to toxicity (Palmer et al., 2019). Studies have confirmed the presence of digoxin-like factors in *Rugosa* Thunb. (Meiguihua), *Flos Hibisci* Mutabilis (Furonghua), and its flavonoids, glycosides, and quercetol (Frész et al., 2014), which cause false-positive results in digoxin assays.

### Enhanced sensitivity of the heart to digoxin and increased risk of toxicity

The main component of *G. glabra* L. (Gancao) is glycyrrhizin, which can be hydrolyzed to glycyrrhizic acid, whose chemical structure is similar to that of corticosterone and which has deoxycorticosterone-like effects, which can “conserve sodium and drain potassium” and cause a sharp decrease in blood potassium levels. Even when digoxin concentrations are within the therapeutic window, digoxin sensitivity increases and digoxin toxicity is easily induced (Dong, 2009). Therefore, it is not advisable to take large amounts of *G. glabra* L. (Gancao) (30–100 g/day) or small amounts of *G. glabra* L. (Gancao) and its preparations orally for a long time, and it is not advisable to combine *G. glabra* L. (Gancao) with digoxin (Chen, 2014)*.* Chinese patent medicines that contain high amounts of *G. glabra* L. (Gancao) include compound glycyrrhiza oral solution/tablet and diammonium glycyrrhizinate enteric-coated capsules.

### Antagonization of digoxin-induced ADR

*Angelica sinensis* (Oliv.) Diels (Danggui), *C. pinnatifida* Bunge (Shanzha), and Calculus Bovis (Niuhuang) can antagonize digoxin-induced arrhythmias. *A. sinensis* (Oliv.) Diels (Danggui) has antiarrhythmic effects and can antagonize digoxin-induced arrhythmias when combined with digoxin (Yang et al., 2019)*.* Li Xin et al. used the duration of QRS interval prolongation, ventricular precontraction, ventricular tachycardia, and ventricular fibrillation as markers of arrhythmia to study the effects of *C. pinnatifida* Bunge (Shanzha) extract on digoxin-induced arrhythmias, and the results showed that the ventricular arrhythmias in the digoxin-induced experimental group of rats all decreased after the administration of *C. pinnatifida* Bunge (Shanzha) extract, indicating that the flavonoid glycoside and flavane polymers in *C. pinnatifida* Bunge (Shanzha) extract can antagonize digoxin-induced arrhythmias (Li and Huang, 2016), which may be related to their ability to dilate blood vessels, lower blood pressure, slow down the heart rhythm, and improve the cardiovascular system (Zhang et al., 2021). 2-Aminoethanesulfonic acid (taurine) is a sulfur-containing non-protein amino acid isolated from Calculus Bovis. In a study in dogs, an initial dose of 35 μg/kg digoxin was administered intravenously to dogs with severed vagus nerves, followed by 17.5 μg every half hour until the electrocardiogram T-wave reversal and premature beats started to show toxic reactions, after which 2-aminoethanesulfonic acid was injected into the femoral vein at 0.5 mmoL/kg every 15 min. The results showed that the electrocardiogram returned to normal, probably because 2-aminoethanesulfonic acid was converted to acyl thiourea intracellularly, reducing the digoxin-induced arrhythmia (Read and Welty, 1963).

In conclusion, TCM interacting with digoxin comprises monomer components, single medicines, and Chinese patent medicines. They can influence the pharmacokinetic process and pharmacodynamics of digoxin, resulting in changes in pharmacokinetic parameters, enhanced efficacy and even poisoning of digoxin. Some of the above changes are clear, while others are speculated to have potential risks through the mechanism of action, which need further study and confirmation. The following table summarizes and analyzes the interaction between Chinese medicine and digoxin from the aspects of the outcome, types of Chinese medicine, research objects, influence mechanism and changes in pharmacokinetic parameters of digoxin (See Table 1).

**TABLE 1:** Interactions between TCM formulations and digoxin, and the underlying mechanisms.

Effect		Drug category or name	Sources of drug/drug composition/example	Study subjects	Mechanism	Pharmacokinetic parameters of digoxin
The effect of digoxin↑	Absorption process affecting pharmacokinetics	Chinese patent medicines containing tropane alkaloids	Belladonna tablets	Premarketing clinical study of digoxin	Decrease intestinal peristalsis, prolong the passage time of digoxin in the upper part of the small intestine Qiu, (2016)	Bioavailability↑
	Transport process affecting pharmacokinetics	Citronellal	*Zanthoxyli Fructus*	*In vitro* cells, Rat Yoshida et al. (2006)	Inhibit gastrointestinal P-gP	Bioavailability↑, CL, V_d_ unchanged
		Nobiletin	Citrus peel	*In vitro* cells, Rat Bai et al. (2018)	Nobiletin and P-gP molecules formed a strong Pi- Pi bond, which inhibited P-gP	AUC↑, C_max_↑
		Quercetin	Flavonoids in plant-based foods, beverages, herbs and dietary supplements	Pig Wang et al. (2004)	Inhibit gastrointestinal P-gP	AUC↑, C_max_↑
		Lanosterin	*Erigeron breviscapus* (Vaniot) Hand.-Mazz.	Mouse Jin et al. (2015)	Inhibit P-gP expression in brain	Not clear, to be further studied
		Cathepsin	One of the polymethoxylated flavonoids in *Artemisia annua*	Rat Yang et al. (2016)	Inhibit P-gP activity and non-competitively inhibit CYP3A	AUC↑, C_max_↑, CL↓
		Citrus juices	Orange juice, lemon juice, grape juice, and grapefruit juice	*In vitro* cells Xu et al. (2003)	Contain a variety of flavonoid components with similar modulatory activity on digoxin transport as P-gP inhibitors	Not clear, to be further studied
		Purple grape	——	*In vitro* cells, Rat Song et al. (2019)	Inhibit gastrointestinal P-gP	AUC↑, C_max_↑
		Ginsenosides	*Panax ginseng* C.A.Mey.	*In vitro* cells (Li et al., 2014)	Inhibit the transport of P-gP to digoxin, compete with digoxin for P-gP binding sites	Plasma concentration↑
		*Conioselinum anthriscoides* 'Chuanxiong'	*Umbelliferae*	*In vitro* cells Yang et al. (2013); Feng et al. (2018)	Inhibit P-gP expression by affecting intracellular Ca^2+^ concentration, bind to P-gP	Plasma concentration↑
			*Conioselinum anthriscoides* 'Chuanxiong'			
		*Vernonia amygdalina* extract	The *Composite* family, *Vernonia amygdalina*	*In vitro* cells, Rat Oga et al. (2012); Oga et al. (2013)	Inhibit P-gP expression	AUC↑
		*Tapinanthus sessilifolius* extract	Roland, *Tapinanthus sessilifolius*	*In vitro* cells, Rat Oga et al. (2012); Oga et al. (2013)	Inhibit P-gP expression	AUC↑
		*Carica papaya* extract	*Caricaceae*, *Carica papaya*	*In vitro* cells, Rat Oga et al. (2012); Oga et al. (2013)	Inhibit P-gP expression	AUC↑
		*Ginkgo biloba* L. extract	*Ginkogo biloba,* *Ginkgo biloba* L.	Rat Rao et al. (2014)	Inhibit gastrointestinal P-gP	Plasma concentration↑
		Wuzhi tablet	*Schisandra chinensis* (Turcz.) Baill.	*In vitro* cells, Mouse Tan et al. (2018)	Inhibit P-gP activity in the liver and kidney, reduce its elimination	Plasma concentration↑
		Shaoyaogancao decoction	*Zhang Zhongjing’s Treatise on Febrile Diseases*, consisting of 2 herbs, *Paeonia lactiflora* Pall. and *Glycyrrhiza glabra* L.	*In vitro* cells Wang et al. (2012)	Downregulate P-gP expression and inhibit P-gP function	Plasma concentration↑, bioavailability↑
	Influence pharmacodynamics (synergy)	*Venenum bufonis* and preparations containing *Venenum bufonis*	The dried white pulp secreted from the skin glands and post-auricular glands of the Chinese toad or the black orbital toad, family Toadidae	*In vitro* Oliveira et al. (2020); Mouse Dasgupta et al. (2002)	They have strong cardioaglyde structures, which can inhibit Na^+^-K^+^-ATPase and block digoxigenin-like effects mediated by voltage-dependent Ⅰ-type calcium channels	Plasma concentration↑
		*Nerium oleander* L.	*Apocynaceae Nerium oleander* L.	*In vitro* cells Amend et al. (2021)	Contain cardiac glycoside components, which are much more toxic than digoxin	Not clear, to be further studied
		*Periploca sepium* Bunge	*Euphorbia* family, *Periploca sepium* Bge.	Mouse Yun et al. (2020)	Periplocymarin has a strong cardiac glycoside structure with strong affinity for Na^+^ -K^+^-ATPase	Not clear, to be further studied
		*Ephedra sinica* Stapf and preparations containing *Ephedra sinica* Stapf	*Ephedra* family *Ephedra sinica* Stapf, *Ephedra intermedia* Schrenk et C.A.Mey. or *Ephedra equisetina* Bge.	Retrospective clinical study Chen, (2014)	Ephedrine has excitatory effects on cardiac α and β receptors, which excite the myocardium and enhance myocardial contractility, and when combined with digoxin, *Ephedra sinica Stapf* can both enhance the cardiotonic effects and the toxic effects of digoxin on the heart, such as arrhythmias and heart failure	Not clear, to be further studied
		TCM of containing Calcium	Qingkailing injection contains mother-of-pearl	Clinical report Dong and Wang, (1993)	It is considered that the mother-of-pearl may contain Ca^2+^ to enhance the effect of cardiac glycosides and its toxicity	Not clear, to be further studied
		Qiliqiangxin capsule	TCM with *Cyperus rotundus* L. *,* *Descurainia sophia* (L.) Webb ex Prantl	Clinical research Ye et al. (2019)	Contain cardiac glycosides, combined with digoxin to strengthen the effect, which is easy to appear cardioside poisoning reaction	Plasma concentration↑
		*Apocynum venetum* L.	*Apocynaceae*, *Apocynum venetum* L.	Dog, Cat Zhai and Lv, (2009)	Contain cardiac glycosides, combined with digoxin to strengthen the effect, which is easy to appear cardioside poisoning reaction	Not clear, to be further studied
		*Tradescantia spathacea* Sw.	Liliaceous plant, *Tradescantia spathacea* Sw.	Clinical report Yu et al. (2012)	Contain cardiac glycosides	Not clear, to be further studied
		Hawthorn weight loss products	*Crataegus pinnatifida* Bunge	Clinical report Palmer et al. (2019)	It has the positive inotropic effect, combined with digoxin to strengthen the effect	Plasma concentration↑
		*Salvia miltiorrhiza* Bunge	*Labiate*, *Salvia miltiorrhiza* Bunge	Clinical research Dasgupta and Szelei-Stevens, (2004)	Contain cardiac glycosides, combined with digoxin to strengthen the effect	Not clear, to be further studied
		*Rugosa* Thunb.	Rosaceous plant, *Rugosa* Thunb.	Clinical research Frész, et al. (2014)	Contain flavonoids such as delphinidin, cyanide and its glycosides, and quercetin, which belongs to digoxin-like factors	Cause false positive and interfere with the determination of digoxin plasma concentration
		*Flos Hibisci* Mutabilis	*Flos Hibisci* Mutabilis	Clinical research Frész, et al. (2014)	Contain flavonoids such as delphinidin, cyanide and its glycosides, and quercetin, which belongs to digoxin-like factor	Cause false positive and interfere with the determination of digoxin plasma concentration
	Affect pharmacodynamics (increase myocardial sensitivity)	*Glycyrrhiza glabra* L. and preparations containing *Glycyrrhiza glabra* L.	Leguminous plants *Glycyrrhiza uralensis* Fisch., *Glycyrrhiza inflata* Bat. or *Glycyrrhiza glabra* L.	Clinical report Dong, (2009)	Hydrolyzes to glycyrrhetinic acid, the chemical structure is similar to corticosterone, which can “retain sodium and expel potassium”, and cause the blood potassium to drop, the sensitivity of cardiac muscle to digoxin is increased	Not clear, to be further studied
The effect of digoxin↓	Absorption process affecting pharmacokinetics	Acidic TCM	Single herb: *Ziziphus jujuba* Mill., *Paeonia lactiflora* Pall., *Schisandra chinensis* (Turcz.) Baill., *Prunus mume* (Siebold) Siebold and Zucc., *Crataegus pinnatifida* Bunge et al.; Chinese patent medicines: Shanzha Pill, Baohe Pill, Wumei Pill et al.	Clinical research Gault et al. (1981)	Gastric acid has a destructive effect on digoxin	Bioavailability↓
		Alkaline TCM	Single herb: sodium borate, decahydrate, *Ostrea gigas* Thunberg; Chinese patent medicines: Bingpeng San, Jianwei Tablet.	Basic study Khalil, (1974)	Elevate the pH of gastric juice, reduce the absorption of digoxin	Bioavailability↓
		TCM with rich fiber	Wheat bran	Clinical research Reissell and Manninen, (1982)	Form a bond with digoxin, which affects the absorption of digoxin	Plasma concentration↓、bioavailability↓
	Transport process affecting pharmacokinetics	Green tea	——	*In vitro* cells Jodoin et al. (2002)	Inhibit P-gP expression	AUC↓, C_max_↓
				Clinical research Kim et al. (2018)		
		MHE	*Radix Bupleurum chinense* DC.	*In vitro* cells, Rat Xiao, (2016)	Increase intestinal P-gP expression	AUC↓, CL/F↑
		*Cyperus rotundus* L. aqueous extract	*Cyperus rotundus* L.	Rat Ji et al. (2019)	Upregulate the expression of P-gP in intestinal tissues from the mRNA and/or protein level	Not clear, to be further studied
		*St. John’s* wort and preparations containing *St. John’s* wort	*Hypericum* family *St. John’s* wort	clinical research Gurley et al., 2008; Dasgupta, (2008); Mueller et al. (2004)	Induce the production of intestinal P-gP	AUC↓, plasma concentration↓
		*Auraptene*	Citrus fruits	*In vitro* cells Nabekura et al. (2020)	Induce drug efflux from the P-gP transporter	Not clear, to be further studied
		*Areca catechu* L. extract	*Areca catechu* L.	*In vitro* cells, Rat Berginc and Kristl (2013)	Induce intestinal P-gP expression	Bioavailability↓
		Shenmai injection	TCM contains *Panax ginseng* C.A.Mey., *Ophiopogon japonicus* (Thunb.) Ker Gawl., *Schisandra chinensis* (Turcz.) Baill.	Dog Mao et al. (2010)	Increase renal excretion	t_1/2_↓, AUC↓
	Influence pharmacodynamics (antagonistic digoxin American depositary receipts)	*Angelica sinensis* (Oliv.) Diels	*Umbelliferae*, *Angelica sinensis* (Oliv.) Diels	Clinical guideline Yang et al. (2019)	It has antiarrhythmic effect, and combined with digoxin can resist the arrhythmia caused by digoxin	Not clear, to be further studied
		*Crataegus pinnatifida* Bunge extract	*Rosaceous* plant, *Crataegus pinnatifida* Bunge	Rat Li and Huang, (2016)	Related to flavonoid glycoside and flavane polymers which can dilate blood vessels, lower blood pressure, slow down heart rhythm and improve the cardiovascular system	Not clear, to be further studied
		2-aminoethanesulfonic acid taurine	Sulfur-containing non-protein amino acid isolated from *calculus bovis*	Dog Read and Welty, (1963)	2-aminoethanesulfonic acid taurine is converted into isothiocyanic acid in cells, which reduces digoxin-induced arrhythmias	Not clear, to be further studied
The effects on digoxin need to be further studied		*Silymarin*	*Silybum marianum* (L.) Gaertn	Retrospective clinical study Wang, (2019)	Induce mRNA expression of P-gP, which may decrease the bioavailability of digoxin and reduce efficacy Wang, (2019), but a clinical research showed no significant effect on pharmacokinetic parameters such as AUC, C_max_, t_1/2_, and CL of digoxin Gurley et al. (2006)	The difference may be related to the compatibility of TCM and the drug users
				Clinical research Gurley et al. (2006)		
		*Panax ginseng* C.A.Mey. and preparations containing *Panax ginseng* C.A.Mey.	*Panax ginseng* C.A.Mey.	Clinical report Li et al. (2011); Chen, (2014); Clinical research Chow et al. (2003)	*Panax ginseng* C.A.Mey. can strengthen the heart and raise blood pressure, some of its molecular structures are similar to digitalis glycosides, which may enhance the effect of digoxin Chen, (2014). However, some studies have shown that ginseng does not affect Chow et al. (2003) or even decrease Li et al. (2011) digoxin plasma concentration	The difference may be related to the source and the compatibility of TCM

## Discussion

Digoxin is a cardiac glycoside drug commonly used in clinical practice for the treatment of heart failure, atrial fibrillation, and other arrhythmias, with a low therapeutic index, a narrow safety range, and large individual differences. It needs to be taken for a long time, and it is often applied in combination with other drugs, especially TCM formulations, in China, which complicates the plasma concentration of digoxin and is prone to poisoning due to the complexity of TCM formulations. In addition, some diets in daily life can also interfere with digoxin plasma concentration. Therefore, clinicians and pharmacists need to understand the interactions between digoxin and TCM formulations to avoid the occurrence of ADRs or even toxic reactions and promote the rational clinical use of drugs. In this paper, we summarize interactions with digoxin and their mechanisms.

**FIGURE 2:**
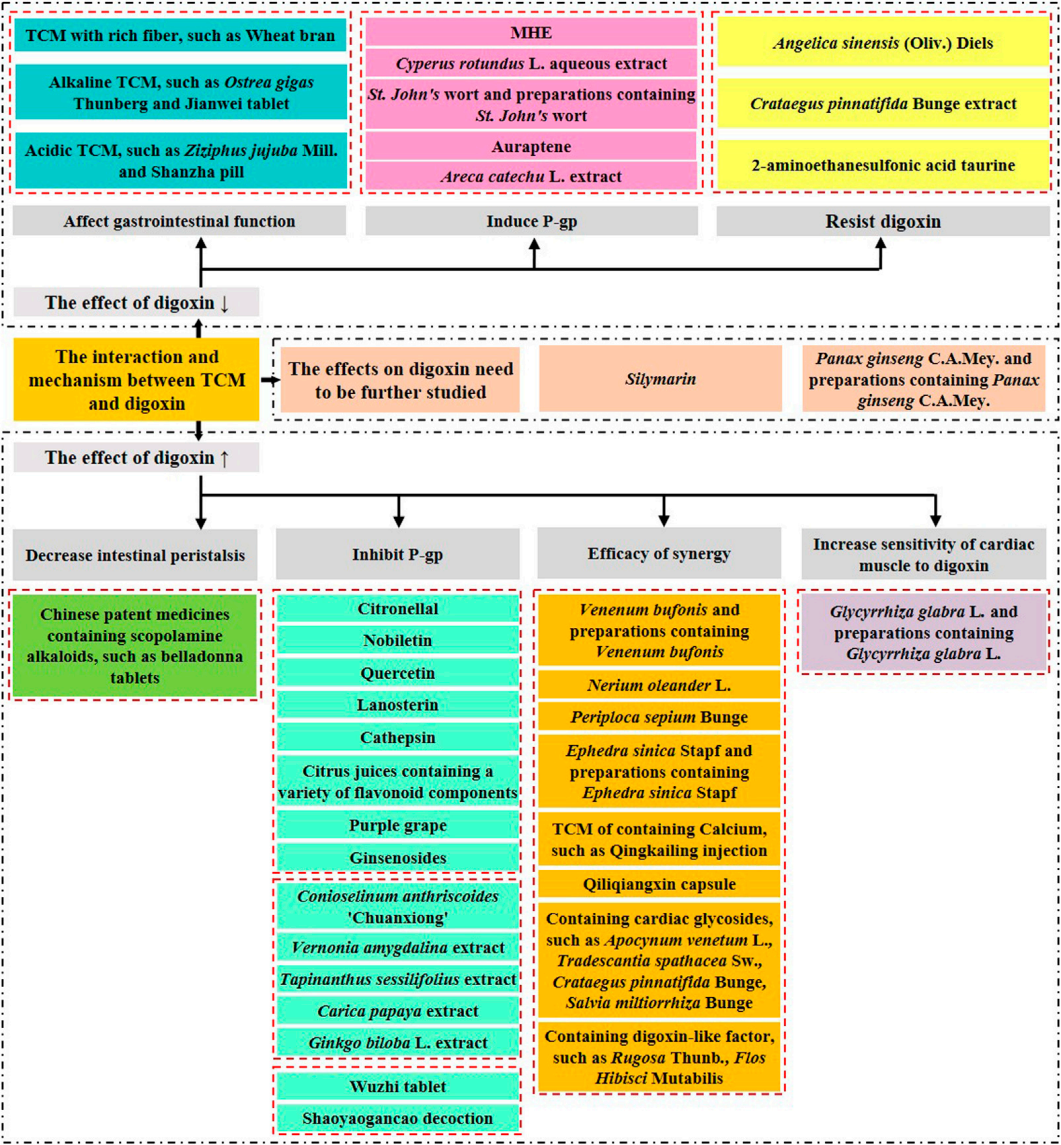
Interactions between TCM formulations and digoxin.

The mechanisms of TCM interactions with digoxin are summarized from the following five perspectives (Figure 2).

*Status of the gastrointestinal tract and digoxin absorption:* TCM formulations containing tropane alkaloids may reduce gastrointestinal motility, delaying absorption and increasing bioavailability; TCM formulations containing large amounts of tannins that cause changes in the pH of the gastrointestinal tract, such as *Z. jujuba* Mill. (Suanzaoren), may lead to a decrease in the bioavailability of digoxin, increasing the risk of peptic ulcers; TCM formulations rich in fiber, such as wheat bran, if ingested at the same time as digoxin, affect the absorption of digoxin, reducing digoxin plasma concentration and bioavailability. Digoxin is combined with TCM which causes the decrease of pH value in the gastrointestinal tract, this view seems to be contradictory to the decrease of pH in gastric juice and the ulcer of the digestive tract when taking digoxin. It is speculated that it is caused by two different analysis angles, and the reasons are as follows: 1) The change of pH caused by the combination with TCM is caused by the change of the external environment, which affects the absorption of digoxin and is caused by external factors. 2) Digestive tract ulcer is easy to occur when taking digoxin, which may be related to the pharmacological effects of digoxin itself. Under normal conditions, the stomach actively secretes acid, and the duodenum actively secretes alkali. The former is driven by H^+^/K^+^-ATPase, while the latter is driven by Na^+^/K^+^-ATPase. Therefore, there is an acid-base balance between the stomach and duodenum. If the balance is broken, it may lead to a peptic ulcer. Digoxin is a selective inhibitor of Na^+^/K^+^-ATPase, which has no inhibitory effect on H^+^/K^+^-ATPase in digestive tract, so it will not inhibit gastric acid secretion. Under normal conditions, Na^+^/K^+^-ATPase coupled with bicarbonate secretion is highly expressed in the stomach and duodenum, so it is speculated that the decrease of pH in the stomach and duodenum is caused by digoxin inhibiting Na^+^/K^+^-ATPase (Fan et al., 2018). Clinical medication should comprehensively consider the acidity and alkalinity of the drugs used, the dosage and course of treatment of digoxin, ask the patient’s medical history, and decide whether to use drugs in combination. If combined, pay close attention to the digestive tract symptoms of patients.

*Regulation of P-gP affecting the digoxin disposal process:* 1) Inhibition or downregulation of P-gP: the monoterpenoid citronellal, flavonoids such as nobiletin, quercetin, lanosterin, and catarrhal xanthin inhibit P-gP activity or suppress P-gP expression, increase plasma concentration of digoxin, and increase bioavailability. Ginsenosides inhibit P-gP transport and compete for P-gP binding sites to increase digoxin plasma concentration. *C. anthriscoides* “Chuanxiong” influences Ca^2+^ concentrations to inhibit P-gP expression and compete for P-gP binding sites to increase digoxin plasma concentration. *V. amygdalina* (Biantaobanjiuju), *T. sessilifolius*, and *C. papaya* inhibit P-gP expression, increase digoxin absorption, and significantly increase the digoxin AUC. *G. biloba* L. (Yinxing) inhibits intestinal P-gP and increases digoxin plasma concentration. Wuzhi Tablet inhibits P-gP activity in the liver and kidney, reduces digoxin elimination, and increases plasma concentration. Shaoyaogancao decoction downregulates P-gP expression, inhibits P-gP function, promotes digoxin absorption, increases plasma concentration and bioavailability. 2) Upregulation of P-gP: silymarin, auraptene, *B. chinense* DC. (Chaihu), *C. rotundus* L. (Fuzi) and *St. John’s* wort can upregulate P-gP and decrease plasma concentration of digoxin. Silymarin induces the mRNA expression of P-gP and decreases the bioavailability of digoxin; *B. chinense* DC. (Chaihu) affects the expression of P-gP, affects the *in vivo* distribution of digoxin, decreases AUC, and increases CL; *C. rotundus* L. (Xiangfu) upregulates the expression of P-gP in intestinal tissues at the mRNA and protein level levels and affects the metabolism of digoxin; *St. John’s* wort activates the P-gP efflux pump, decreases plasma concentration of digoxin, and decreases AUC; auraptene induces P-gP to pump out the drug. 3) Others: Flavonoids contained in citrus fruits and purple grapes inhibit P-gP activity or suppress P-gP expression, increase plasma concentration of digoxin, and increase bioavailability. Green tea inhibits P-gP and reduces the pharmacokinetic parameters of digoxin such as AUC and C_max_. *A. catechu* L. (Dasuan) decreases bioavailability of digoxin.

Medicinal and edible homologous substances are edible and medicinal Chinese herbal medicines used in TCM and diet therapy based on the guidance of the concept of homologous medicine and food. The application history has a long history, which is recorded in “*Shen Nong’s Classic of the Materia Medica*”, “*The Grand Compendium of Materia Medica*” and other books. For example, the ancients used edible bark in nature and processed it into food to replace food to satisfy hunger, which played a role in satisfying and curing diseases. The buds, seedlings, leaves, or flowers of medicinal plants with the homology of medicine and food in wood are processed into drinks, which have good taste, and can play a role in healthcare and conditioning discomfort symptoms after long-term use. With the rapid development of social economy, modern life makes people have higher expectations and pursuit for physical and mental health. Medicinal varieties with the homology of medicine and food should be paid attention to. Most medicinal plants with the homology of medicine and food are gentle in nature, warm in nature and sweet in taste. When the bias of medicine is greater than that of food, it will inevitably affect the balance of qi, blood, yin and yang of the human body (Jia et al., 2021). The homology of medicine and food does not mean that medicine and food are the same. In practical application, special attention should be paid to the difference between medicine and food. Excessive or improper consumption can easily lead to toxicity (bias). While knowing its edible value, we should also pay attention to its medicinal value, so as to avoid drug interaction and affect the treatment of diseases.

*Synergistic drug effects increase the risk of digoxin poisoning:* Venenum bufonis has a cardiac glycoside structure, inhibits Na^+^/K^+^-ATPase, blocks voltage-dependent L-type calcium channels to exert cardiac effects, enhances digoxin plasma concentration, and causes toxic reactions; *N. oleander* L. (Jiazhutao) and *P. sepium* Bunge (Xiangjiapi) contain cardiac glycoside components that inhibit Na^+^/K^+^-ATPase; *P. ginseng* C.A.Mey (Renshen) has some molecular structures that are similar to digitalis glycosides; *E. sinica* Stapf (Caomahuang) and preparations containing *E. sinica* Stapf excite cardiac α and β receptors and enhance the cardiotonic effects of digoxin; calcium-containing TCM formulations increase serum calcium ion concentrations and inhibit Na^+^/K^+^-ATPase, which can synergistically enhance the effect of digoxin; Qiliqiangxin capsules have components that contain cardiotonic glycosides, i.e., *C. rotundus* L. (Fuzi) and *D. sophia* (L.) Webb ex Prantl (Tinglizi). Other cardiac glycoside-containing TCM formulations, such as *A. venetum* L. (Luobuma), *T. spathacea* Sw (Wannianqing), and *S. miltiorrhiza* Bunge (Danshen), when combined with digoxin, increase the risk of cardiac glycoside toxicity due to cumulative pharmacological effects; TCM formulations containing digoxin-like factors such as *Rugosa* Thunb. (Meiguihua) and *Flos Hibisci* Mutabilis (Furonghua) interfere with the determination of digoxin plasma concentration and may cause false-positive results. Qiliqiangxin capsules combined with digoxin, the risk of increasing digoxin blood concentration and even poisoning is related to age, patients over 80 years old are more likely to be poisoned by the increase of digoxin blood concentration, which may be due to the decrease of renal function and creatinine clearance with the increase of age, resulting in the higher digoxin plasma concentration. Therefore, the digoxin plasma concentration of elderly patients should be monitored to avoid poisoning (Ye et al., 2019).

*Enhanced sensitivity of the heart to digoxin and increased risk of toxicity: G. glabra* L. (Gancao) has deoxycorticosterone-like effects, which can “conserve sodium and drain potassium,” easily causing hypokalemia, increasing the sensitivity of myocardium to digoxin, and inducing digoxin toxicity in therapeutic doses.

*Antagonize digoxin-induced ADR: A. sinensis* (Oliv.) Diels (Danggui) has anti-arrhythmic effects; *C. pinnatifida* Bunge (Shanzha) extract contains flavonoid glycosides and flavane polymers that can dilate blood vessels, lower the blood pressure, and slow down the heart rhythm; 2-aminoethanesulfonic acid is converted to acyl thiourea intracellularly, reducing the cellular response to digoxin-induced arrhythmia. All the above herbs can antagonize the arrhythmic toxic reactions induced by digoxin.

Although a lot of experience has been accumulated in the clinical application of digoxin over the years, limited research has been conducted on its possible interactions with TCM formulations. The mechanisms of interaction between TCM formulations and digoxin have been basically elucidated in terms of pharmacokinetics and pharmacodynamics in cells, animals, and humans. Based on the reported pharmacological effects and existing research results, some Chinese herbal medicines may have potential interaction with digoxin. The following problems were found in the present analysis: i) Existing studies on the effects of TCM formulations on P-gP mainly focus on a certain component or a single drug, while few studies have been conducted on Chinese patent medicines containing these components. ii) Studies have been conducted on the interactions of TCM extracts with digoxin, but the specific components of the extracts were not identified. For example, a study showed that a small molecule component of the water-soluble part of *B. chinense* DC. (Chaihu) has an induction effect on P-gP, but its water-soluble part contains a variety of monosaccharides, oligosaccharides, amino acids, and other components, and it is not clear which component causes the interaction. iii) The results of the interactions between Chinese patent medicines, TCM formulations, and digoxin are inconsistent. For example, *P. ginseng* C.A.Mey. enhances the effect of digoxin, but Shenmai injection (a Chinese patent medicine containing *P. ginseng* C.A.Mey.) promotes the renal excretion of digoxin and decreases digoxin plasma concentration; *G. biloba* L. (Yinxing) has no effect on the pharmacokinetic parameters of digoxin, while *G. biloba* L. extract has a significant effect and increases digoxin plasma concentration. These discrepancies may be related to the formulation of the Chinese patent medicine, the dose, the assay method, and other factors; further study is necessary. iv) The pharmacological effects of TCM formulations are complex, and most of the current studies have been conducted in cells and animals. Whether TCM formulations that can regulate P-gP also interfere with human digoxin plasma concentration needs further clinical validation.

In view of the above problems in the research of the interaction between TCM and digoxin, the following solutions are put forward: 1) Because of the complex components of TCM, the pharmacology of some TCMs has not been clarified. It is suggested that network pharmacology should be used to clarify the components and mechanism of TCM in the future. 2) For Chinese medicines found to have exact or potential interaction with digoxin, it is suggested to study from different levels of monomer, single medicine, ready-for-use TCM or prescription in the future to confirm whether the action with digoxin is related to a certain component, a certain medicine, mutual compatibility, processing and other factors. 3) At present, most of the research data come from animal experiments or cells. For TCMs that have interaction found *in vitro* studies, it is suggested to further conduct clinical research to provide basis for the safe combination of digoxin and TCMs. 4) The combination of Chinese and Western medicine is not only simply adding Chinese medicine with Western medicine, but it should also be a combination of the theoretical basis and scientific medication principles. It is suggested that clinicians, while ensuring clinical efficacy, should scientifically and reasonably select digoxin to combine with TCM, monitor the blood concentration of digoxin, record the patient information, the dosage, course of treatment and combination of digoxin, and establish a database of the combination of digoxin and TCM, so as to timely find out the TCM that may interact with digoxin, provide data support, and play a warning role in the clinical drug combination.

Among the factors affecting the pharmacokinetics and pharmacodynamics of digoxin, special population (elderly people, people with abnormal renal function), genes and races are mentioned, mainly considering that the safety range of digoxin itself is narrow, and there are many factors affecting the blood concentration of digoxin, among which special population, genes and races will also affect the blood concentration of digoxin. Because of the decrease of renal blood flow, glomerular filtration rate and renal excretion function in the elderly, the blood concentration of digoxin increased obviously. Digoxin mainly passes through glomerular filtration and is excreted in its original form in human body. Because of the decrease of glomerular filtration rate and the prolongation of half-life, the blood concentration of digoxin is significantly increased in patients with renal insufficiency, and it is easy to appear poisoning (Xia, 2017).

The research has demonstrated that genes (Jiang et al., 2019) and ethics (Yan et al., 2015) have an impact on the blood concentration of digoxin, which reminds patients to consider the individualization of clinical medication and ask the basic information of patients carefully, which provides a new angle for the study of blood concentration of digoxin. For patients with abnormal digoxin blood concentration or poisoning which are difficult to be explained by existing research theories, gene detection, gene sequencing, race and geographical origin of patients can be considered, which may provide new influencing factors for the safe use of digoxin in the future.

Considering the narrow therapeutic window of digoxin, the effective therapeutic dose is not easy to determine, and it is prone to poor efficacy or toxic reactions (Englund et al., 2004). P-gP inhibitors applied in combination are positively correlated with patients’ digoxin plasma concentration. Therefore, when combining digoxin with one or more P-gP inhibitors, clinicians or pharmacists should consider the patient’s condition, the TCM components, the interactions between digoxin and TCM formulations, and the compatibility between drugs. Moreover, the plasma concentration of digoxin should be monitored to pay close attention to its efficacy and toxicity and to the interaction between digoxin and TCM formulations in clinical applications, with a view to develop better treatment options and to guide the rational and safe use of clinical drugs.

## Conclusion

With the popularization of TCM and the improvement of living standards, elderly patients who have been taking digoxin for a long time tend to take TCM on their own. Digoxin has a narrow safety range, i.e., the therapeutic and toxic doses are close to each other, and it is associated with large individual differences. Drug interactions have a great effect on digoxin plasma concentration and cause toxic reactions. The composition of TCM formulations is complex. Therefore, the interactions between TCM formulations and digoxin are difficult to elucidate, and they are easily ignored by physicians. When patients take digoxin, they should take into account other medications and digoxin plasma concentration should be monitored to ensure efficacy and safety. Doctors and pharmacists should screen patients for the TCM formulations mentioned in this paper that have interactions with digoxin, monitor the use of interacting drugs such as those containing flavonoids or calcium, those affecting P-gP, and herbal medicines with cardiac potency, and adjust the type and dose of medication according to the monitoring results and clinical symptoms to prevent or reduce the occurrence of ADRs and improve efficacy and safety.

## Abbreviations

ABC, ATP-binding cassette transporter; ADR, adverse reaction; AUC, area under the curve; C_max_, maximum plasma concentration; CL, clearance rate; MHE, micromolecular hydrophilic extract from vinegar baked Radix Bupleuri; pH, potential of hydrogen; P-gP, P-glycoprotein; RIA, radioimmunoassay; t_1/2_, half-life period; TCM, traditional Chinese medicine; V_d_, apparent volume of distribution.
